# Histological Study of a Novel 3D-Printed Hydroxyapatite/PLGA Bone Graft in the Regeneration of Critical-Sized Long Bone Defects

**DOI:** 10.3390/bioengineering13040394

**Published:** 2026-03-28

**Authors:** Marijana Popović Bajić, Smiljana Paraš, Milutin Mićić, Božana Petrović, Vladimir Biočanin, Slavoljub Živković, Marija Živković, Damjana Drobne, Vukoman Jokanović

**Affiliations:** 1 School of Dental Medicine, University of Belgrade, 11000 Belgrade, Serbia; marijana.popovic@stomf.bg.ac.rs (M.P.B.); slavzivkovic@gmail.com (S.Ž.);; 2 Faculty of Science and Mathematics, University of Banja Luka, 78000 Banja Luka, Bosnia and Herzegovina; smiljana.paras@pmf.unibl.org; 3 Ortoprint d.o.o., 14000 Valjevo, Serbia; 4 Institute of Nuclear Sciences Vinča—National Institute of the Republic of Serbia, University of Belgrade, 11351 Belgrade, Serbia; vjokan@gmail.com; 5 Faculty of Dentistry in Pančevo, University of Business Academy Novi Sad, 26000 Pančevo, Serbia; vladimirbiocanin@gmail.com; 6 Biotechnical Faculty, University of Ljubljana, 1000 Ljubljana, Slovenia; damjana.drobne@bf.uni-lj.si; 7 ALBOS d.o.o., 11000 Belgrade, Serbia

**Keywords:** nano hydroxyapatite, rabbit model, bone reconstruction, bone regeneration, segmental osteotomy, 3D printing

## Abstract

Critical-sized bone defects pose significant challenges in orthopedic surgery. The introduction of 3D printing technology in bone grafting offers a promising solution by creating customized grafts that mimic the natural bone structure. This study aimed to reconstruct long-segment bone defects in the rabbit radius using a 3D-printed material composed of hydroxyapatite (HAP) and poly(lactide-co-glycolide) (PLGA), referred to as ALBO-OS, and to evaluate its potential to support bone healing without the use of stem cells or growth factors. Six rabbits underwent computed tomography scanning to create patient-specific 3D models of the radius. Custom-designed ALBO-OS implants were 3D-printed and used to fill segmental defects corresponding to one-third of the bone length in each rabbit, created by osteotomy. Over a 12-week observation period, graft integration, osteointegration, and overall bone regeneration were assessed through histological and histomorphometric analyses. The implanted scaffolds demonstrated encouraging bone healing, with significant bone regeneration observed within the defect areas. Histological evaluation revealed significant new bone formation and vascularization, with minimal inflammatory response. The findings demonstrated the potential of 3D-printed HAP/PLGA-based materials as a promising strategy for the reconstruction of large bone defects, eliminating the need for exogenous biological agents.

## 1. Introduction

Bone tissue engineering is an exciting and rapidly evolving field dedicated to developing materials that can effectively replace damaged or lost bone tissue while seamlessly integrating with the surrounding biological environment [1]. The primary goal of bone substitute materials is twofold: they must provide immediate mechanical stability at the defect site while simultaneously promoting natural bone regeneration. Ultimately, the objective is to restore the original biomechanical function of the affected bone [2].

For a bone substitute to be truly effective, it must exhibit several key physical and functional properties [3]. Structurally, the material should closely resemble natural bone in terms of compressive strength, toughness, and porosity. Studies suggest that an ideal bone substitute should possess a porosity greater than 50% and an average pore size exceeding 100 μm to facilitate efficient cellular infiltration, vascularization, and nutrient exchange [4]. Additionally, the material must be osteoconductive, enabling cell migration and nutrient transport; osteoinductive, stimulating the formation of new bone; and osteointegrative, ensuring stable and long-term integration with the surrounding bone tissue [5,6]. Another critical factor influencing the success of bone regeneration is the surface microstructure of the material at both the micro- and nano-scales [7]. This surface structure plays a crucial role in directing cell adhesion, proliferation, and differentiation, making it an essential component of modern bone substitute design.

One of the major challenges in the field is the treatment of critical-sized bone defects—defects that are too large to heal spontaneously through the body’s natural regenerative mechanisms [8]. These defects present significant difficulties due to factors such as their size, anatomical location, and the complexity of the required surgical procedures. Given these challenges, 3D printing has emerged as a powerful tool for the development of patient-specific bone substitutes, enabling highly customized designs that optimize both structural integrity and functional recovery [9,10,11,12].

A key determinant of success in bone substitute materials is the balance between new material resorption and new bone formation [13]. If the material degrades too quickly, it may not provide adequate support before new bone tissue has fully developed. Conversely, if the material resorbs too slowly, it can disrupt the remodeling process and lead to implant failure [14]. Current bone substitute materials, including calcium sulfate, hydroxyapatite (HAP), and coral-derived biomaterials, have notable limitations. Calcium sulfate exhibits a high solubility rate and therefore degrades too quickly, sometimes before new bone has a chance to form. HAP-based materials degrade too slowly, which may cause them to persist in the body long after their intended function has been fulfilled. Coral-derived bone substitutes closely resemble the structure of natural bone but are typically brittle and unable to withstand significant mechanical stress [15].

A promising alternative to these existing materials is a novel HAP and poly(lactide-co-glycolide) (PLGA)-based composite material, named ALBO-OS. This next-generation bioactive material has been engineered to provide ideal mechanical properties, controlled degradation rates, and osteogenic potential, without genotoxic effect, making it highly suitable for bone regeneration [16,17,18]. Studies have demonstrated its effectiveness, including complete healing of rabbit calvarial defects within just 12 weeks [18].

Despite major advancements in bone regeneration research [19], most studies have focused on partial bone resection, in which a segment of bone is removed without completely disrupting its continuity. Tang et al. were the first to report full-thickness bone reconstruction, where an entire bone segment was excised, severing both the proximal and distal ends [20]. In their study, stem cells were seeded onto the scaffold prior to implantation, and bone morphogenetic protein 2 (BMP-2) was incorporated to enhance osteogenesis. However, one of the ultimate goals of modern bone tissue engineering is to develop fully synthetic, bioresorbable bone substitutes that support natural bone regeneration without the need for additional biological enhancers, such as stem cells or growth factors [21]. In a previous pilot study, our research group successfully demonstrated the feasibility of this approach by reconstructing a rabbit ulna using a 3D-printed HAP–PLGA-based graft [22]. A key limitation of that study, however, was the use of only a single experimental animal. Therefore, the present study builds upon our earlier work and aims to further evaluate the effectiveness of this graft design in a larger experimental scope.

The primary aim of this study was to reconstruct long-segment bone defects (equivalent to one-third of the total bone length) in the rabbit radius using a 3D-printed, bioresorbable, and osteoinductive ALBO-OS material. The focus was on achieving successful bone healing without stem cells or growth factors, demonstrating that engineered bone substitutes could be effective for large-scale defect reconstruction. The null hypothesis of this study is that no significant bone regeneration occurs at the defect site in the presence of an implant.

## 2. Materials and Methods

The HAP applied in this study was synthesized using a hydrothermal method, following established protocols [18]. Briefly, the synthesis process involved preparation of aqueous solutions of calcium hydroxide (Ca(OH)_2_) and ammonium phosphate ((NH_4_)_2_HPO_4_) (Merck KGaA, Darmstadt, Germany). The two solutions were combined and autoclaved at 120 °C for 2 h to ensure phase purity. The synthesized HAP particles were filtered through a 200 nm membrane to achieve a uniform particle size. To further enhance the material’s properties, a thin PLGA coating was applied. PLGA (50:50, M = 45,000–70,000; Durect Corporation, Cupertino, CA, USA) was dissolved in chloroform and deposited onto the HAP particles. After solvent evaporation, a uniform PLGA film remained on the particles’ surface. So, the obtained bone substitute, named ALBO-OS, was further used for 3D printing of a bone implant according to the corresponding 3D model of the rabbit radius.

**Figure 1:**
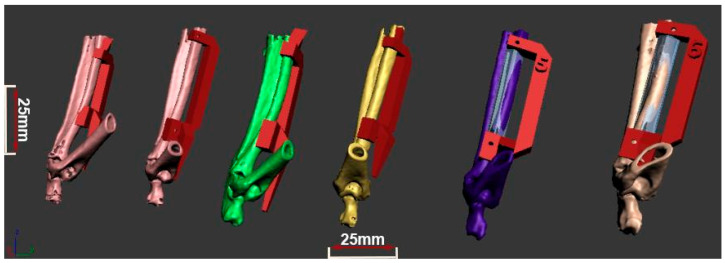
Planning of the surgical guides for defect induction and restoration by bone grafts for the 6 rabbit models involved. 3D models of the rabbit’s long bones are given in different colors for specimens 1–6 (from left to right). Surgical guides of the 3D models, given in red color, were used for 3D printing of the surgical guides that were used (fixated by pins on planned positions) for inducing the bone defect and fixing the remaining bone ends in the correct position for the application of the bone graft. The bone graft is shown in the transparent blue color on the part of the radius that was planned for restoration in specimens 5 and 6.

**Figure 2:**
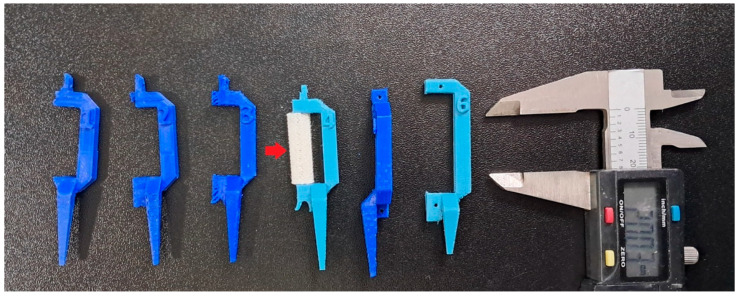
3D-printed individual surgical guides with fixation holes (blue color) and 3D-printed bone grafts made from ALBO-OS and PLGA (white color, red arrow pointing to it).

A total of six New Zealand white rabbits underwent pre-operative and postanesthetic computed tomography (CBCT) scanning of the radius using a Scanora 3D scanner (Soredex, Tuusula, Finland). The imaging parameters included: 13 mA current, 90 kV voltage, and 0.2 mmv voxel resolution. CT scan data were processed using the Slicer 4.3.1 software (Brigham and Women’s Hospital, Boston, MA, USA) and Blender 4.3 (Stichting Blender Foundation, Amsterdam, The Netherlands) to create detailed 3D models of the rabbit radii. These models were then refined in Autodesk 3D Max 2010 (Autodesk Inc., San Francisco, CA, USA). In all 6 cases, the defect on the rabbit radius was planned using Autodesk 3D Max software 2010, in such fashion that the cutting plane was planned perpendicular to the long bone, approximately 10 to 20 mm distal to the proximal radius end (Figure 1), and the complete diameter of the radius was removed in approximately 1/3 of the radius length (the resulting defect measured 25 mm in length and 7 mm in diameter). For every animal involved, an individual surgical guide was 3D-planned and 3D-printed (Figure 1), along with 3D planning and printing of the bone graft (Figure 2).

The 3D-printed bone implants were fabricated using a custom-built 3D printer (Albos d.o.o., Belgrade, Serbia), based on the original Prusa I3 design (Prusa Research, Praha, Czech Republic) [23]. The Pronterface software 2.0.1. was used for precise 3D printing of the bone substitutes, using a paste made of ALBO-OS particles less than 300 µm and PLGA as a support material (Figure 2). Printing parameters were: layer height—0.20 mm, bed temperature—60 °C, nozzle temperature—200 °C, infill density—15%, and print speed—50 mm/s. The implant was fabricated using a gradient approach, with the ALBO-OS concentration gradually increasing toward the implant’s center (from 20 to 80%) to ensure both mechanical stability and controlled resorption rates. Accordingly, the maximum concentration of PLGA was achieved on the construct surface. Once printed, the 3D bone implants underwent 12 h of UV sterilization to eliminate any potential microbial contamination before implantation.

The in vivo experiment involved six adult New Zealand white rabbits, each aged four months old and weighing approximately 2 kg. The study followed the EU guidelines (86/609/EEC) for the ethical use of laboratory animals and was approved by the Ethics Committee of the Faculty of Veterinary Medicine, University of Belgrade (Approval No. 323-07-08477/2015-05/3, issued on 8 March 2016).

To ensure a smooth surgical procedure, the rabbits were administered intramuscular premedication, consisting of Ketamidor (ketamine hydrochloride, 10%), 35 mg/kg (Richter Pharma Ag, Wels, Austria), and Xylased (xylazine, 2%), 5 mg/kg (Bioveta, Ivanovice na Hané, Czech Republic). For pain management, Butorphanol (0.1 mg/kg, intramuscularly, Richter Pharma Ag, Wels, Austria) was given. Additionally, lidocaine hydrochloride (2%) (Galenika a.d., Belgrade, Serbia) was applied at the surgical site to provide local anesthesia. Before surgery, each rabbit underwent pre-operative CBCT scanning of the left radius to obtain detailed anatomical data. These imaging results were used to design patient-specific 3D bone models, ensuring perfect alignment with the bone defect.

A 35 mm linear incision was made along the left radius, and the overlying soft tissues were carefully elevated to expose the bone. A 25 mm-long osteotomy was performed, removing one-third of the total bone length in each rabbit. Once the bone segment was removed, the custom 3D-printed bone implant was precisely implanted into the defect without using stem cells or growth factors. The defect was strategically placed at a predetermined distance from the proximal radius to ensure proper anatomical alignment. To confirm the accurate positioning of the implant, mediolateral X-ray imaging was performed using a ZooMax White DR system 1.0 (Control-X Medical, Ltd., Dunakezsi, Hungary), with the following imaging parameters: 47 kV voltage, 6.4 mAs current, and 70 cm focal-film distance. The X-ray images were digitized using a CR 10-X scanner (Agfa HealthCare NV, Mortsel, Belgium) for further analysis. Once the correct placement of the bone implant was verified, the soft tissues were repositioned, and the surgical wound was closed using simple interrupted sutures (Vicryl 3-0, Ethicon, Raritan, NJ, USA).

After surgery, the rabbits were housed in separate cages, with free access to food and water. They were closely monitored for pain, infection, and overall well-being. During the first five days post-surgery, no signs of pain, distress, or loss of appetite were observed. To ensure pain relief and infection prevention, the following medications were administered: Buprenorphine (Alkaloid AD, Skopje, Republic of North Macedonia) (0.1 mg/kg, twice daily for five days) for post-operative pain management and Oxytetracycline (Dopharma B.V., Zalmweng, The Netherlands) (20 mg/kg, subcutaneous) to prevent bacterial infections. Pain levels were evaluated using the Rabbit Grimace Scale (RGS), a standardized method for assessing facial expressions linked to pain. Throughout the recovery period, no significant discomfort was detected. Sutures were removed ten days post-surgery, and the incision site was carefully examined. There were no signs of swelling, infection, or wound dehiscence, indicating smooth healing.

At the 12-week post-surgery mark, X-ray imaging was performed again, after which all six rabbits were humanely euthanized, and both radius bones (left and right) were carefully harvested for histological and morphological analysis. A 12-week period was selected as it allows sufficient time for the initiation and progression of key physiological processes involved in damaged tissue regeneration, enabling meaningful assessment of regeneration. The right radius served as a control (untreated bone), and the left radius, which contained the 3D bone implant, was examined to evaluate bone regeneration and biocompatibility. Immediately after removal, the bone samples were fixed in 4% neutral buffered formalin to preserve tissue integrity for detailed analysis.

To assess bone regeneration, the rabbit radii were sectioned 10 mm proximal and 10 mm distal to the implant site for sample collection. The collected bone samples were subjected to standard light microscopy analysis, using the following methodology: (1) fixation: immediately after collection, all samples were preserved in 4% buffered formaldehyde for a month to maintain tissue structure; (2) decalcification: bone samples were treated with formic acid to soften mineralized tissue for sectioning; (3) dehydration: samples were gradually dehydrated using increasing concentrations of ethanol; and (4) paraffin embedding: the processed samples were embedded in paraffin wax for sectioning.

Longitudinal 0.004 mm-thick sections were prepared and stained with hematoxylin and eosin (H&E) to evaluate overall tissue morphology, picrosirius red for collagen staining and immunohistochemical staining to detect osteocalcin expression (a key marker of bone formation), and Masson’s trichrome dye, which enables better visualization of soft connective tissue, as well as reticular and collagenous tissue.

A detailed quantitative evaluation of bone regeneration was performed in both the central and peripheral regions of the defect. Four tissue sections were analyzed per sample, with 0.05 mm spacing between sections. Morphometric analysis was conducted at 400× magnification, using a digital camera (Leica DFC295, Wetzlar, Germany). Histological analysis was performed using light microscopy (Leitz Labor Lux S fluorescent microscope, Ernst Leitz Wetzlar GMBH, Wetzlar, Germany), while histomorphometric analysis was conducted using a software package (Leica University Suite, version 4.3, Leica Microsystems, Wetzlar, Germany).

Stereological analysis was performed using a universal stereological test system based on Cavalier’s principle, employing a 16.0 point-counting system, MBF Application Suite 3.0.0. software (MBF Bioscience, Williston, VT, USA). Measurements were performed using a P2 spacing grid under a microscope at a maximum magnification of ×400. Micrographs were initially captured in RGB format and subsequently converted to binary images for stereological assessment.

The evaluated stereological parameters of the bone tissue included: volume density of osteocytes, volume density of bone matrix number, volume density of blood vessels in bone tissue, number of osteocytes, numerical density of osteocytes, surface area of osteocytes, surface area of osteocytes’ nuclei, the nucleocytoplasmic ratio (NCR) of osteocytes, and blood vessel diameter.

H&E-stained sections of radius bone tissue were used for determining the numerical density (Nv) and total number of osteocytes per unit volume of cells. The volume density (Vv) of blood vessels was calculated using the formula Vv = Pf/Pt (mm0), where Pf represents the number of test points matching the desired phase (all blood vessels), and Pt is the total number of test points.

Micrographs of bone tissue were analyzed in binary format (black and white) to quantify the number and numerical density of osteocytes. Points were counted according to the following criteria: osteocyte nuclei were considered reference points; surface area measurements included only cells with visible contours and nuclei that did not touch the test frame; and cells with contours but without visible nuclei within the test system were also included. The numerical density (Nv) of all osteocytes was calculated using the formula:

$$Nv=\frac{Q}{V_{0}}=\frac{Q}{\sum_{i=1}^{n}P_{i}\times a\times h}\;({\mathrm{mm}}^{-3})$$

where *Q* is the number of counted cells, *V_0_* is the volume of the analyzed tissues, Σ*Pi* is the number of analyzed frames, *a* is the area of the counting frame (a = 0.025 mm^2^), and *h* is the section thickness (0.004 mm).

The surface areas and volumes of osteocytes and their nuclei were estimated based on measured diameters using the MBF software 2024.1.3. The nucleocytoplasmic ratio (NCR) was defined as the ratio between the nuclear volume and cytoplasmic volume of the osteocytes.

All measurement results were expressed as mean values accompanied by standard deviations. Depending on the distribution of the data, group comparisons were conducted using either parametric (*t*-test) or nonparametric (Mann–Whitney U-test) methods. A *p*-value below 0.05 was considered statistically significant. Statistical analyses were carried out using SPSS software, version 20.0 (IBM Corp., Armonk, NY, USA).

## 3. Results

**Figure 3:**
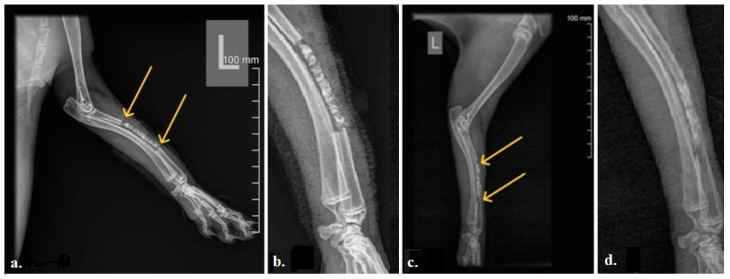
Representative radiogram of the rabbit radius: (**a**) Immediately after implantation of the bone graft. We can see an ideal fitting of the graft into the defect (direct contact of the graft edges with the proximal and distal ends of the previously sectioned bone defect, yellow arrows). (**b**) Enlarged area of the implant immediately after implantation. (**c**) 12-weeks post-operative. Complete bone healing at the place of the 3D-printed bone graft near the surrounding bone ends can be noticed (yellow arrows). Also, higher bone density inside the 3D-printed bone graft was noticed. (**d**) Enlarged area of the implant after 12 weeks.

The radiograms of the rabbit radii 12 weeks after implantation showed that the efficiency of bone tissue regeneration in the rabbit radius is lower in the central part of the defect compared to the peripheral areas that were in contact with the old bone (Figure 3). The yellow arrows in Figure 3c point out the complete bone healing at the contact site of the 3D-printed bone graft and old bone, with no radiolucency between the graft and the surrounding bone. Additionally, increased bone density, reflected in a more homogeneous bone structure within the 3D-printed graft compared with the initial radiogram, is noticeable.

**Figure 4:**
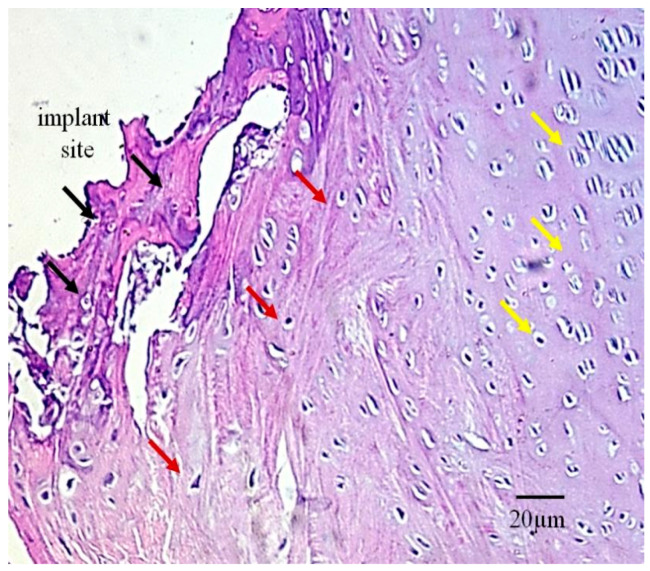
A typical appearance of newly formed bone and regenerated surrounding tissue near the implant–bone interface 12 weeks after implantation: the black arrows indicate irregularly shaped chondrocytes occupying the space between the defect and the implant (implant site), the red arrows mark chondrocytes involved in regeneration and healing of the defect, which are not yet fully organized into a normal cartilage cytoarchitecture, and the yellow arrows indicate completely healthy and structurally regular cartilage tissue (Masson’s trichrome stain, magnification 400×).

**Figure 5:**
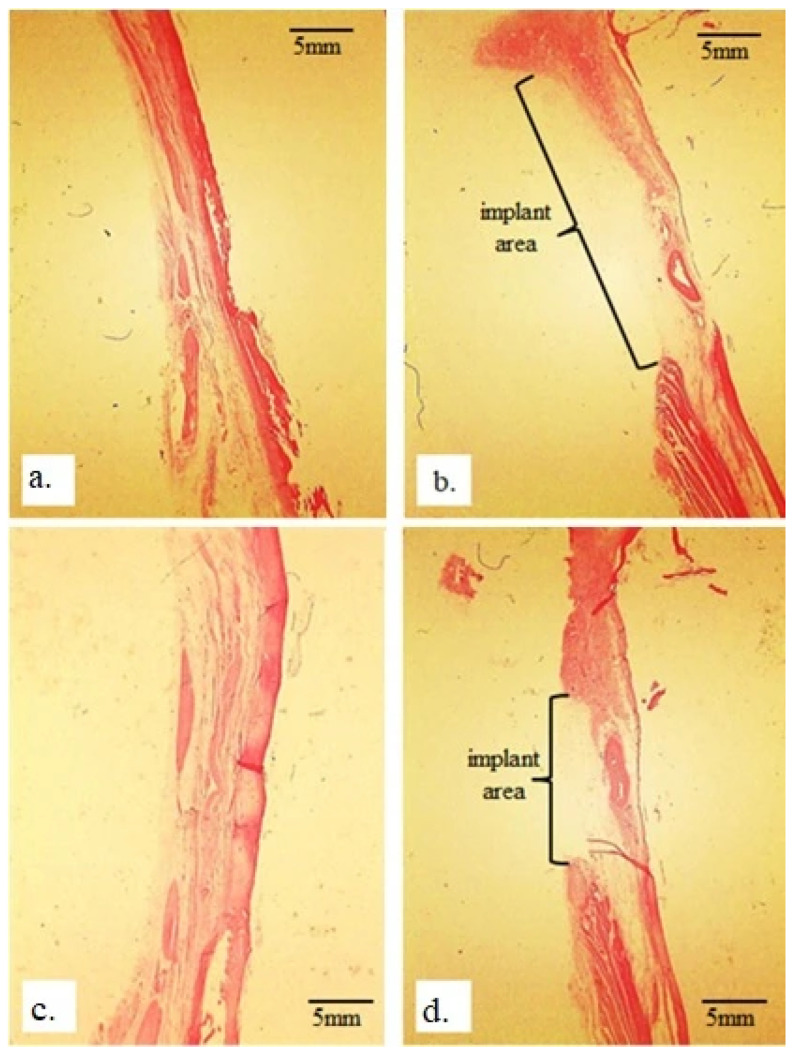
Micrographs of longitudinal sections of rabbit radius: (**a**,**c**) without an implant and (**b**,**d**) with a 25 mm implant; wherein (**a**,**b**) represent immunohistochemical staining using the Picrosirius red technique and (**c**,**d**) represent H&E staining (magnification 10×).

**Figure 6:**
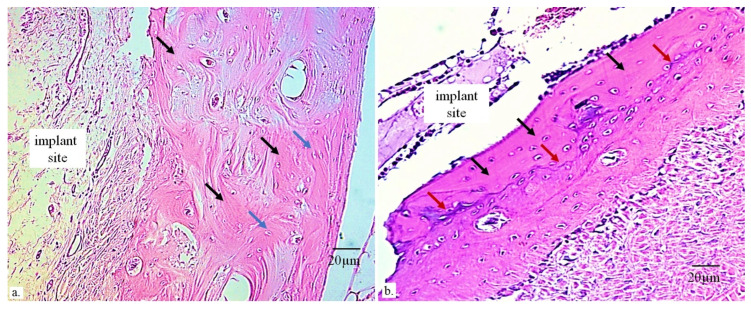
(**a**) Osteocalcin immunostaining demonstrating new bone formation. Layers of extracellular matrix around the osteoblasts secreting osteocalcin (black arrows) appear as parallel, wave-like lines, indicating ongoing ossification. Osteoblasts (blue arrows) remain osteogenically active and continue forming new bone. Areas with stronger pink coloration correspond to regions of active osteogenesis (i.e., bone tissue regeneration), while regions with more intense blue coloration contain less osteocalcin, indicating immature bone. (**b**) Clusters of osteocalcin, showing stronger immunoreactivity in the thin, newly formed bone adjacent to the implant (black arrows), are produced by osteoblasts (red arrows). The bluish line, along which the red arrows are aligned, marks the boundary with the older bone that was damaged during implantation and is undergoing regeneration, characterized by prominent blood vessels (large circles). (Immunohistochemical staining using the Picrosirius red technique, 200× magnification).

**Figure 7:**
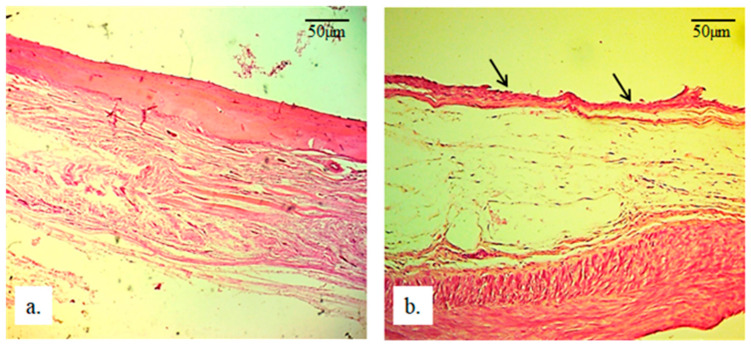
Micrographs of longitudinal sections of rabbit radius: (**a**) without an implant and (**b**) with a 25 mm implant. Black arrows indicate newly formed bone (H&E staining, magnification 100×).

**Figure 8:**
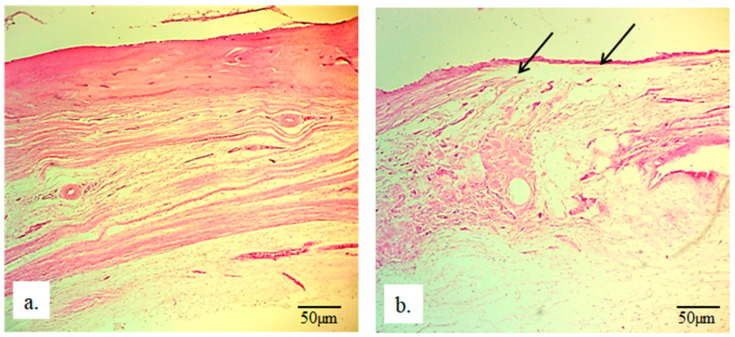
Micrographs of longitudinal sections of rabbit radius: (**a**) without an implant and (**b**) with a 25 mm implant. Black arrows indicate newly formed bone (H&E staining, magnification 100×).

**Figure 9:**
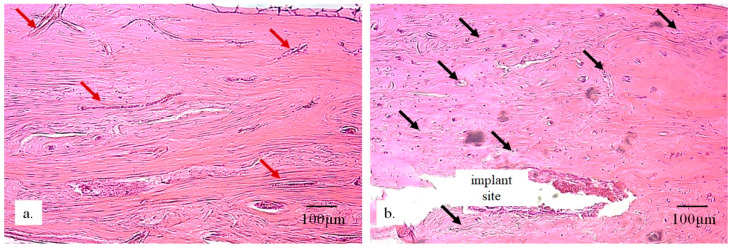
Cross-section of bone tissue: (**a**) control sample and (**b**) sample with the implant, showing vascularization: the presence of larger blood vessels (red arrows) and smaller blood vessels—capillaries, arterioles, and venules (black arrows) (Immunohistochemical staining using the Picrosirius red technique, magnification 200×).

**Figure 10:**
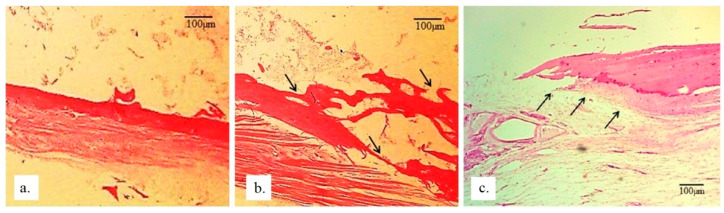
Micrographs of longitudinal sections of rabbit radius: (**a**) without an implant; (**b**,**c**) with a 25 mm implant. Black arrows indicate newly formed bone filling the implant space, wherein (**b**) represents immunohistochemical staining using the Picrosirius red technique and (**c**) represents H&E staining (magnification 200×).

Therefore, both bone tissue regions were analyzed using histomorphometric and stereological parameters. Stereological (Table 1) and histomorphological (Figure 4, Figure 5, Figure 6, Figure 7, Figure 8, Figure 9 and Figure 10) analyses of longitudinal sections of rabbit radius revealed the presence of newly formed bone tissue without a statistically significant difference in quality compared to the bone tissue of the control rabbit radius. A statistically significant difference was observed only in the volume density of capillaries in the newly formed bone within the implant compared with the control group (*p* = 0.0209). This finding primarily reflects the predominance of small blood vessels in the newly formed (younger) tissue, in contrast to the larger, more mature vessels (venules and arterioles) present in the control group. The newly formed bone tissue in the treated rabbit radii exhibited lower volumes of osteocytes and blood vessels, while the volume of the extracellular matrix was increased. Differences in these values of these parameters confirmed the presence of osteogenesis in the regions of the radius containing the implant. Additionally, standard histomorphometric measurements were performed: the percentage of newly formed bone area within the defect (14.71 ± 2.88%), the percentage of residual graft material (60.77 ± 7.92%), and the area of bone–implant contact (85.25 ± 10.55%).

After 12 weeks of implant presence within the bone defect, the bone tissue consisted not only of newly formed bone but also of regenerated surrounding bone that had been subjected to cutting trauma during defect creation (Figure 4). New bone formation was observed in the narrow space between the implant surface and the intentionally created defect surface, appearing as irregularly arranged bone cells and extracellular matrix (Figure 4, black arrows). Additionally, the previously damaged bone surface—characterized by disrupted blood vessels and crushed osteocytes—showed clear signs of regeneration over the 12-week period (Figure 4, red arrows).

Figure 5 illustrates the differences in the morphology of the rabbit radius without implants (Figure 5a,c) and those with implants (Figure 5b,d). The newly formed bone tissue in the radius bones is very thin and fragile, considering that the defect size of 25 mm represents approximately one-third of the total length of the rabbit radius. The newly formed bone tissue is in close contact with the old bone in the peripheral part of the defect, with a very subtle demarcation line between them. Immunohistochemical staining for osteocalcin and collagen confirmed the presence of calcium and collagen throughout the rabbit radius and on its surface, which is in contact with the implant (Figure 6).

Figure 5d shows the space in the rabbit radius where the implant was located, compared to the healthy bone without an implant (Figure 5c). By comparing Figure 5b,d, a difference can be seen; in the first image (Figure 5b), the space is wider and less defined, whereas in the second one (Figure 5d), the space seems deeper and shorter. This indicates that the bone tissue of the radius is undergoing regeneration in both the peripheral and central parts of the bone, surrounding the implant from all sides (Figure 6). These results suggest a positive effect of bone tissue after implant placement, regardless of the implant size, which accounted for approximately one-third of the total length of the rabbit radius.

At the junction between the implanted material and the radius bone, no lymphocytes are present (Figure 7); only a small number of fibroblasts can be observed under the newly formed bone (Figure 7b). The newly formed bone is extremely thin (black arrows), with young and numerous osteocytes. Additionally, in the bone tissue of the radius with the implant, no cysts, necroses, infiltration of any type of blood cells, or immune response cells are observed.

In Figure 8b, the black arrows indicate the newly formed bone of the rabbit radius and its thin appearance compared to the same bone in radii without an implant (Figure 8a). Below the thin newly formed bone, irregular reticular newly formed connective tissue can be observed, which also exhibits a different architecture compared to the same tissue in the radius without an implant. Additionally, the presence of blood capillaries in this tissue is crucial, as they enable further regeneration, vitality, and functionality of the newly formed tissues (Figure 9).

Figure 9 shows evident infiltration of newly formed blood capillaries and the development of new blood vessels (black arrows). The bone tissue with implants contains a higher number of capillaries—which is clearly visible in the image—while the control tissue contains a greater proportion of larger, differentiated, fully formed blood vessels.

The detection and visualization of collagen were performed using immunohistochemical Picrosirius red staining, which binds to collagen in the extracellular matrix of newly formed bone. In Figure 10, a very strong immunoreactivity to collagen is observed, indicating ongoing osteogenesis processes in the newly formed bone in the radius with implants. In the same image, lamellar formation of new bone is visible, along with an irregular arrangement (black arrows in Figure 10b) where spaces still contain reticular tissue that has not yet been replaced by bone. Additionally, immunohistochemical staining that detects osteocalcin in the bone tissue demonstrated that the newly formed bone is highly mineralized (Figure 10b). The largest amount of newly formed bone shows signs of lamellar organization, with viable osteocytes concentrically positioned around Haversian canals. In addition to the mineralized bone, there are areas of non-mineralized newly formed bone, directly indicating the presence of active osteogenesis. The presence of immature (woven) bone suggests processes of active bone tissue remodeling. The newly formed bone predominantly has a trabecular architecture, with new bone marrow present between the trabeculae.

In Figure 10c, the presence of chondrocytes in hyaline cartilage is observed, indicating both direct and indirect ossification. The newly formed cartilage is present in a very thin layer (black arrows), composed of irregularly organized and young chondrocytes. The precise location of the hyaline cartilage chondrocytes within the defect is visible in Figure 4, and they differ from one another in their organization and cytoarchitecture. Newly formed cartilage has extended between the defect surface and the implant, as it will later give rise to young bone. The spatial dynamics and progression of bone regeneration occur from the defect surface toward the implant. In the early phase, the young chondrocytes are irregularly shaped and thread-like, and lighter and darker purple regions of stained extracellular matrix can be observed around them (around the black arrows, Figure 4), indicating that collagen synthesis is not yet fully completed.

The architecture of the newly formed tissue in the radius of rabbits with 25 mm implants (Figure 10b) is altered compared to the same tissue in the control radius of rabbits (Figure 10a). The numerical density of osteocytes is lower in the bone tissue of the rabbit radius that had implants, compared to the control group (Table 1). Additionally, both the surface area of osteocytes and the size of their nuclei are smaller and irregular in shape (Figure 10c) in the tissue of radii with implants, compared to the control ones. In all radii with implants, pronounced resorption of the implant is observed, and in that space, new bone and connective tissue are forming, with some particles of bone substitute incorporated into the newly formed bone tissue of the radius. The bone tissue of the rabbit radius with the implant shows a very good level of regeneration and repair, as indicated by the high NCR of osteocytes (Table 1). The presence of fibroblasts and fibrocytes in the newly formed bone tissue suggests the presence of processes related to the acceptance of the implant by the bone tissue of the rabbit radius, as well as the ongoing processes of its regeneration and repair.

All the results presented above demonstrate significant new bone formation and vascularization, accompanied by minimal inflammatory response, indicating successful bone healing in the absence of stem cells or growth factors. These findings suggest that engineered bone substitutes can be effective for the reconstruction of large-scale bone defects.

## 4. Discussion

The obtained results of histological and histomorphometric analyses conducted at 12-weeks post-implantation revealed successful bone regeneration at the implant site, confirming that the 3D-printed bone implant served as an effective scaffold for new bone formation. Peripheral regions showed the development of mature lamellar bone, with Haversian osteons and well-formed bone marrow spaces, while central regions exhibited osteoblast infiltration and early-stage mineralization, signifying active osteogenesis.

The findings of this study are consistent with those of Tang et al. [20], who demonstrated significant bone regeneration using bioactive glass enriched with recombinant human BMP-2 (rhBMP-2). Tang et al. [20] reported 10% new bone formation with bioactive glass alone and 40% bone regeneration with the addition of BMP-2 growth factors. In contrast, our study achieved substantial bone regeneration without the use of stem cells or growth factors, emphasizing the potential of engineered bone substitutes with intrinsic osteoinductive properties.

Our study highlights how the engineered construct acts as a three-dimensional (3D) template, mimicking the natural architecture of bone tissue. Its design supports bone cell attachment, growth, movement, and the formation of new bone. The construct’s porosity, analyzed in our previous study [22], meets key structural requirements (>50% total porosity and >100 μm average pore size), making it effective for ion exchange and maintaining homeostasis [12,24]. The interconnected pores allow efficient nutrient and oxygen flow, which is vital for cell survival and differentiation in bone tissue engineering [25]. Additionally, the scaffold provides mechanical stability, preventing early collapse and ensuring structural support throughout the bone regeneration process [26]. Although mechanical testing was not performed in the present study and is planned for future work, radiographs of the rabbit radii at 12 weeks post-implantation showed no displacement of the osteomized bone and demonstrated complete healing at the interface between the 3D-printed bone graft and the native bone, which indicates that the graft provided sufficient structural stability to support normal healing.

The approach applied in this research eliminates the need for stem cells and growth factors by creating a bone substitute with natural osteoinductive properties. This represents a major leap in bone tissue engineering, shifting the focus from external biological enhancements to the material’s intrinsic capabilities.

Despite numerous bone replacement materials developed in the past decade, none have fully met the needs for repairing large bone defects [27,28,29]. Many materials lack sufficient mechanical support and the ability to synchronize degradation of implanted material with new bone formation [6,15,30]. As a result, achieving full bone repair in the center of the construct remains difficult, often leading to fibrous tissue formation and inadequate vascularization. This study addressed these challenges, successfully reconstructing one-third of the rabbit ulna using two key technological advancements.

First, the construct was 3D-printed using a nano HAP-based material with a critical advantage: its dissolution rate closely matches that of natural bone. This balance ensures that the surrounding bone has enough time to regulate bone growth and material breakdown without leading to fibrous tissue formation at the interface. By closely mimicking natural bone degradation, the construct allows for a seamless transition, preventing stress shielding and improving long-term stability. Notably, in the central region of the construct, where new bone is typically formed in the final stages of healing, the material gradually transformed into a slightly lamellar structure.

The second major innovation in the present study was the successful 3D printing of the HAP-based scaffold, which achieved both the necessary mechanical strength and the high porosity required for bone formation. The scaffold’s composition, along with its mechanical properties and specific 3D structure, provided multiple biochemical and physical signals that influenced cellular behavior and controlled the timing of bone regeneration. The combination of material composition, structural design, and physicochemical properties highlights the construct’s potential as a next-generation bone substitute for clinical use.

Our findings confirm that the 3D-printed bone implant acts as a scaffold, closely mimicking the structural properties of natural bone tissue. It supports cell migration, attachment, proliferation, and new bone formation, creating an ideal environment for bone regeneration. The use of a specialized nano HAP material with a PLGA coating significantly improved bone healing, allowing for the successful reconstruction of a large radius bone defect. This innovative method tackles a major challenge in orthopedic tissue engineering by aiding the repair of large bone defects, which are often difficult to heal due to poor blood supply and limited oxygen flow at the defect site.

In a previous pilot study, we successfully reconstructed rabbit ulna using a 3D-printed nHAP-based graft that was precisely tailored to match the anatomical shape and size of the defect [22]. The nHAP material, designed to degrade at a rate similar to natural bone, facilitated proper healing while preventing the growth of fibrous tissue, which could otherwise hinder bone regeneration. However, a limitation of this study was the use of only a single experimental animal. Before in vivo testing, the biocompatibility of the 3D-printed graft was rigorously assessed in direct contact with SCAPs, confirming that it was non-toxic [22]. Moreover, the graft actively encouraged cell migration and bone cell differentiation, further proving its potential for clinical applications. Detailed histological analysis revealed osteoblast migration into the 3D-printed graft, while newly formed blood vessels supported bone growth in surrounding areas. Additionally, the printed nHAP-based graft not only provided essential mechanical support but also featured the necessary porosity to enhance bone integration. The combination of the graft’s material properties, structural characteristics, and mechanical strength appears to play a critical role in delivering key physical and chemical signals that influence cell behavior and the timing of regeneration.

## 5. Conclusions

In conclusion, our study highlights the immense potential of 3D-printed bone grafts for the regeneration of large bone defects, marking a significant step forward in orthopedic tissue engineering. After 12 weeks of healing, substantial bone regeneration was observed, with mature mineralized bone forming at the defect margins and active bone formation continuing within the defect area. These findings suggest that 3D-printed bone grafts may represent a promising alternative to traditional grafting methods, particularly for the treatment of large and complex bone defects. However, further research is required to validate these results in larger animal models and to assess the long-term stability and integration of the regenerated bone. In addition, future studies should focus on strategies to improve blood supply in order to enhance bone regeneration in the central regions of the defect. Nevertheless, our results demonstrate the promising potential of 3D-printed bone grafts in regenerative medicine, offering a viable approach for repairing extensive bone defects without the need for stem cells or growth factors. With continued research and technological refinement, this approach may contribute significantly to future clinical strategies for bone reconstruction and orthopedic treatments.

## Figures and Tables

**Table 1:** Stereological parameters of bone tissue analysis in rabbit radii with 25 mm implants.

Parameter	Control (*n* = 6)	Implanted Bone (*n* = 6)
Volume density of osteocytes (mm^0^)	0.275 ± 0.016	0.231 ± 0.013
Volume density of bone matrix (mm^0^)	0.582 ± 0.039	0.648 ± 0.072
Volume density of lamellar bone tissue (mm^0^)	0.684 ± 0.055	0.662 ± 0.051
Volume density of regenerated bone tissue (mm^0^)	0.126 ± 0.011	0.149 ± 0.017
Volume density of blood vessels in bone tissue (mm^0^)	0.143 ± 0.011	0.121 ± 0.011
Volume density of capillaries in the intact and regenerated bone (mm^0^)	0.082 ± 0.009	0.099 ± 0.009
Volume density of capillaries in the newly formed bone (mm^0^)	0.023 ± 0.005	0.057 ± 0.005 *
Number of osteocytes	27,336.5 ± 1903.8	22,731.4 ± 1795.6
Numerical density of osteocytes (mm^−3^)	2682.9 ± 388.8	2467.5 ± 362.4
Surface area of osteocytes (mm^2^)	1.566 × 10^−4^ ± 1.22 × 10^−5^	1.253 × 10^−4^ ± 1.15 × 10^−5^
Surface area of osteocytes’ nuclei (mm^2^)	6.57 × 10^−5^ ± 9.8 × 10^−6^	5.89 × 10^−5^ ± 9.2 × 10^−6^
Nucleocytoplasmic ratio (NCR) of osteocytes	0.375 ± 0.039	0.409 ± 0.041
Diameter of blood vessels (mm)	0.0456 ± 0.0049	0.0495 ± 0.0041

* Statistically significant difference between groups for *p* < 0.05.

## Data Availability

The original contributions presented in this study are included in the article. Further inquiries can be directed to the corresponding author.

## Abbreviations

The following abbreviations are used in this manuscript:

3D	Three dimensional
HAP	Hydroxyapatite
PLGA	Poly(lactide-co-glycolide)
ALBO-OS	Name of material based on nHAP and PLGA
CBCT	Cone beam computed tomography
H&E	Hematoxylin and eosin
NCR	Nucleocytoplasmic ratio

## References

[1] Koushik T.M., Miller C.M., Antunes E. (2022). Bone Tissue Engineering Scaffolds: Function of Multi-Material Hierarchically Structured Scaffolds. Adv. Healthc. Mater..

[2] Rizwan M., Hamdi M., Basirun W.J. (2017). Bioglass^®^ 45S5-based composites for bone tissue engineering and functional applications. J. Biomed. Mater. Res. A.

[3] Chen S., Wang M. (2026). Fabrication, Properties, and Applications of Scaffolds for Bone Tissue Regeneration. Adv. Mater. Technol..

[4] Xu C., Su P., Chen X., Meng Y., Yu W., Xiang A.P., Wang Y. (2011). Biocompatibility and osteogenesis of biomimetic Bioglass-Collagen-Phosphatidylserine composite scaffolds for bone tissue engineering. Biomaterials.

[5] Yao Q., Cosme J.G., Xu T., Miszuk M.J., Picciani P., Fong H., Sun H. (2017). Three dimensional electrospun PCL/PLA blend nanofibrous scaffolds with significantly improved stem cells osteogenic differentiation and cranial bone formation. Biomaterials.

[6] Sohn H.S., Oh J.K. (2019). Review of bone graft and bone substitutes with an emphasis on fracture surgeries. Biomater. Res..

[7] Mohammadi M., Shaegh S.A.M., Alibolandi M., Ebrahimzadeh M.H., Tamayol A., Jaafari M.R., Ramezani M. (2018). Micro and nanotechnologies for bone regeneration: Recent advances and emerging designs. J. Control. Release.

[8] Huang E.E., Zhang N., Shen H., Li X., Maruyama M., Utsunomiya T., Gao Q., Guzman R.A., Goodman S.B. (2022). Novel Techniques and Future Perspective for Investigating Critical-Size Bone Defects. Bioengineering.

[9] Lee K.-G., Lee K.-S., Kang Y.-J., Hwang J.-H., Lee S.-H., Park S.-H., Park Y., Cho Y.-S., Lee B.-K. (2018). Rabbit Calvarial Defect Model for Customized 3D-Printed Bone Grafts. Tissue Eng. Part. C Methods.

[10] Damiri F., Fatimi A., Musuc A.M., Santos A.C.P., Paszkiewicz S., Idumah C.I., Singh S., Varma R.S., Berrada M. (2024). Nano-Hydroxyapatite (nHAp) Scaffolds for Bone Regeneration: Preparation, Characterization and Biological Applications. J. Drug Deliv. Sci. Technol..

[11] Noory P., Farmani A.R., Ai J., Bahrami N., Bayat M., Ebrahimi-Barough S., Farzin A., Shojaie S., Hajmoradi H., Mohamadnia A. (2025). Enhancing in vitro osteogenic differentiation of mesenchymal stem cells via sustained dexamethasone delivery in 3D-Printed hybrid scaffolds based on polycaprolactone-nanohydroxyapatite/alginate-gelatin for bone regeneration. J. Biol. Eng..

[12] Komlev V.S., Popov V.K., Mironov A.V., Fedotov A.Y., Teterina A.Y., Smirnov I.V., Bozo I.Y., Rybko V.A., Deev R.V. (2015). 3D printing of octacalcium phosphate bone substitutes. Front. Bioeng. Biotechnol..

[13] Yang B.C., Lee J.W., Ju C.P., Chern Lin J.H. (2020). Physical/Chemical Properties and Resorption Behavior of a Newly Developed Ca/P/S-Based Bone Substitute Material. Materials.

[14] Cho J.H., Song H.K. (2025). Current concepts and applications of bone graft substitutes in orthopedic surgery. J. Musculoskelet. Trauma.

[15] Yao Q., Wei B., Guo Y., Jin C., Du X., Yan C., Yan J., Hu W., Xu Y., Zhou Z. (2015). Design, construction and mechanical testing of digital 3D anatomical data-based PCL-HA bone tissue engineering scaffold. J. Mater. Sci. Mater. Med..

[16] Paraš S., Trišić D., Mitrović Ajtić O., Prokić B., Drobne D., Živković S., Jokanović V. (2020). Toxicological Profile of Nanostructured Bone Substitute Based on Hydroxyapatite and Poly(Lactide-Co-Glycolide) after Subchronic Oral Exposure of Rats. Nanomaterials.

[17] Mitić D., Čarkić J., Jaćimović J., Lazarević M., Jakšić Karišik M., Toljić B., Milašin J. (2024). The Impact of Nano-Hydroxyapatite Scaffold Enrichment on Bone Regeneration In Vivo—A Systematic Review. Biomimetics.

[18] Jokanović V., Čolović B., Marković D., Petrović M., Soldatović I., Antonijević D., Milosavljević P., Sjerobabin N., Sopta J. (2017). Extraordinary Biological Properties of a New Calcium Hydroxyapatite/Poly(Lactide-Co-Glycolide)-Based Scaffold Confirmed by in Vivo Investigation. Biomed. Eng. Biomed. Tech..

[19] Sabouri Z., Dequeecker M., Anees H., Adib F.R., Jamous R., Zheng J., Lyu X., Stoetzel S., Heiss C., El Khassawna T. (2026). Recent advances in biomaterials for bone regeneration: Bridging innovation and clinical translation. Mater. Today Bio.

[20] Tang W., Lin D., Yu Y., Niu H., Guo H., Yuan Y., Liu C. (2016). Bioinspired trimodal macro/micro/nano-porous scaffolds loading rhBMP-2 for complete regeneration of critical size bone defect. Acta Biomater..

[21] Checherita L.E., Chirilov A.M., Parvu S. (2025). Bioresorbable Scaffolds in Bone Tissue Engineering. Med. Mater..

[22] Micic M., Antonijevic D., Milutinovic-Smiljanic S., Trisic D., Colovic B., Kosanovic D., Prokic B., Vasic J., Zivkovic S., Milasin J. (2020). Developing a novel resorptive hydroxyapatite-based bone substitute for over-critical size defect reconstruction: Physicochemical and biological characterization and proof of concept in segmental rabbit’s ulna reconstruction. Biomed. Eng. Biomed. Tech..

[23] Paraš S., Petrović B., Mitić D., Lazarević M., Popović Bajić M., Živković M., Mićić M., Biočanin V., Živković S., Jokanović V. (2025). Three-Dimensional-Printed Bone Grafts for Simultaneous Bone and Cartilage Regeneration: A Promising Approach to Osteochondral Tissue Engineering. Pharmaceutics.

[24] Swanson W.B., Omi M., Woodbury S.M., Douglas L.M., Eberle M., Ma P.X., Hatch N.E., Mishina Y. (2022). Scaffold Pore Curvature Influences ΜSC Fate through Differential Cellular Organization and YAP/TAZ Activity. Int. J. Mol. Sci..

[25] Mukasheva F., Adilova L., Dyussenbinov A., Yernaimanova B., Abilev M., Akilbekova D. (2024). Optimizing scaffold pore size for tissue engineering: Insights across various tissue types. Front. Bioeng. Biotechnol..

[26] Todd E.A., Mirsky N.A., Silva B.L.G., Shinde A.R., Arakelians A.R.L., Nayak V.V., Marcantonio R.A.C., Gupta N., Witek L., Coelho P.G. (2024). Functional Scaffolds for Bone Tissue Regeneration: A Comprehensive Review of Materials, Methods, and Future Directions. J. Funct. Biomater..

[27] NicNicolae C.-L., Pîrvulescu D.-C., Niculescu A.-G., Epistatu D., Mihaiescu D.E., Antohi A.M., Grumezescu A.M., Croitoru G.-A. (2024). An Up-to-Date Review of Materials Science Advances in Bone Grafting for Oral and Maxillofacial Pathology. Materials.

[28] Schulze F., Lang A., Schoon J., Wassilew G.I., Reichert J. (2023). Scaffold Guided Bone Regeneration for the Treatment of Large Segmental Defects in Long Bones. Biomedicines.

[29] Wang Z., Lv Z., Cai X., Wang Y., Peng B., Xu H., Pang H., Yang X., Xu J., Bian Y. (2026). Sculpting the Future of Bone: The Evolution of Absorbable Materials in Orthopedics. Adv. Mater..

[30] Xia B., Liu Y., Xing Y., Shi Z., Pan X. (2025). Biodegradable Medical Implants: Reshaping Future Medical Practice. Adv. Sci..

